# Age-Related Platelet Cox-1 Upregulation in Atrial Fibrillation

**DOI:** 10.3390/ijms27114972

**Published:** 2026-05-30

**Authors:** Emanuele Valeriani, Vittoria Cammisotto, Danilo Menichelli, Daniele Pastori, Valentina Castellani, Simona Bartimoccia, Giovanni Cucchiara, Arianna Pannunzio, Ilaria Maria Palumbo, Ombretta Martinelli, Roberto Carnevale, Francesco Violi, Pasquale Pignatelli

**Affiliations:** 1 Department of Internal Medicine, Endocrine and Metabolic Sciences, and Infectious Diseases, Azienda Ospedaliero-Universitaria Policlinico Umberto I, 00161 Rome, Italy; emanuele.valeriani@outlook.com; 2 Department of Medical and Cardiovascular Sciences, Sapienza University of Rome, 00161 Rome, Italy; vittoria.cammisotto@uniroma1.it (V.C.); daniele.pastori@uniroma1.it (D.P.);; 3 Department of General Surgery and Surgical Specialty, Sapienza University of Rome, 00161 Rome, Italy; 4 Scientific Institute for Research, Hospitalization and Healthcare IRCCS Neuromed, 86077 Pozzilli, Italy; 5 Internal Medicine, Sapienza University of Rome, 00161 Rome, Italy; 6 Department of Surgery “Paride Stefanini”-Policlinico Umberto I, Vascular Surgery Division, Sapienza University of Rome, 00161 Rome, Italy; ombretta.martinelli@uniroma1.it; 7 Department of Medical-Surgical Sciences and Biotechnologies, Sapienza University of Rome, 04100 Latina, Italy

**Keywords:** aging, aspirin, atrial fibrillation, cyclooxygenase 1, thromboxane

## Abstract

Aging is associated with enhanced platelet activation that may contribute to the occurrence of cardiovascular events. However, the mechanism linking aging with platelet activation is not fully understood. The objective of this study is to investigate the relationship between aging, platelet Cox-1 expression, and thromboxane (Tx) B2 production in patients with atrial fibrillation. Serum Cox-1 and TxB2 were measured in 134 patients with atrial fibrillation. Correlations were assessed between age, Cox-1, and TxB2. A robust mediation analysis evaluated whether Cox-1 mediates the association between age and TxB2. In vitro experiments were performed in 20 patients to evaluate the effect of aspirin on platelet TxB2 production and to quantify platelet Cox-1 expression across age groups (i.e., < or ≥65 years). Serum Cox-1 and TxB2 progressively increased by decades of age. A positive and significant correlation was found between age and Cox-1 (R = 0.42, *p*-values < 0.01), age and TxB2 values (R = 0.44, *p*-value < 0.01), and Cox-1 and TxB2 (R = 0.5, *p*-value < 0.01). Cox-1 partially and significantly mediated the effect of age on TxB2 (β = 5.23, 95% confidence interval [2.33–8.63]) for the effect of age on TxB2. In vitro analysis showed a reduced inhibitory effect of aspirin on platelet TxB2 production in old compared to young subjects (IC_50_ 97 μM and 49 μM in ≥ and <65 years, respectively) that was paralleled by Cox-1 overexpression in patients ≥65 years. Platelet Cox-1 expression was inversely related with aspirin inhibitory effects (R = −0.640, *p*-value < 0.01). Aging is associated with a concomitant increase in Cox-1 concentration and TxB2 production and an impaired ability of aspirin to inhibit Cox-1.

## 1. Introduction

Aging is associated with a substantial residual risk of cardiovascular events and atherothrombosis, even in the context of contemporary optimal medical therapy [1,2]. This residual vulnerability appears to be particularly pronounced in specific patient subgroups, such as individuals with atrial fibrillation, in whom standard antithrombotic regimens do not fully mitigate the age-related prothrombotic milieu [3,4]. Furthermore, the prescription of antithrombotic agents, such as aspirin, necessitates a careful risk–benefit analysis, given that the increased susceptibility to hemorrhage in older patients can outweigh the marginal antithrombotic efficacy [5].

Among the mechanisms proposed to explain this age-dependent phenomenon, platelet activation and aggregation represent a key contributor [3,6,7]. Recent evidence indicates that platelets from older individuals undergo structural and signaling alterations that increase their susceptibility to activation and reduce inhibitory responsiveness, potentially fostering a hyperreactive platelet phenotype that may contribute to the development of an age-related prothrombotic state [8]. Consistently, persistent biosynthesis of thromboxane (Tx) A2 has been associated with aging, and urinary levels of 11-dehydro-TxB2—a stable TxA2 metabolite—increase with age and may predict the risk of atherothrombotic events or cardiovascular death in aspirin-treated patients [9,10]. Similar observations have been reported in patients with atrial fibrillation in whom a progressive age-related increase in urinary 11-dehydro-TxB2 has been documented, potentially implicating platelet activation pathways in the heightened vulnerability to cardiovascular events observed with aging [3,4].

Nevertheless, the specific mechanisms driving platelet Tx overproduction in older adults remain poorly understood. We hypothesized that this phenomenon may reflect an age-related upregulation of platelet cyclooxygenase (Cox)-1, the enzyme catalyzing arachidonic acid conversion to TxA2 [11]. To address this, we examined whether serum Cox-1 levels increase with age and are linked to the age-associated rise in serum TxB2. In parallel, to complement our clinical findings, we performed in vitro experiments in a randomly selected subpopulation of our cohort to (1) evaluate aspirin’s effect on platelet TxB2 production in relation to aging, (2) to quantify platelet Cox-1 expression across age groups, and (3) to assess the correlation between platelet Cox-1 expression and the rate of the inhibitory effect of aspirin on platelet TxB2 production.

## 2. Results

### 2.1. Baseline Characteristics of Patients

Baseline characteristics of included patients are reported in Table 1. Overall, 134 patients with atrial fibrillation were included in the primary analysis with a median age of 75 years (IQR, 67 to 80 years) and a 43.3% proportion of females. Arterial hypertension was present in 89.6% of patients, diabetes mellitus in 38.7% of patients, and dyslipidemia in 51.5% of patients. Roughly half of the patients were former smokers (45.3%) and 12.3% were active smokers. A history of carotid atherosclerotic disease was present in 32.1%, coronary artery disease in 15.1%, and cerebrovascular disease in 18.9% of patients. Median CHA_2_DS_2_-VA score was 3 (2–4) and median HAS-BLED score was 1 (1–1).

Most of the included patients received direct oral anticoagulants (76.9%) compared to vitamin K antagonists (i.e., warfarin; 23.1%). Regarding concomitant therapies, ACE-inhibitors or angiotensin receptor blockers were administered in 69.8% of patients, beta-blockers in 66.0% of patients, and statins in 64.2% of patients. No patients received concomitant antiplatelet therapy.

Mean hemoglobin value was 13.7 g/dL (± 1.3 g/dL), median platelets 212,000/mm^3^ [IQR, 170,000 to 246,000/mm^3^], median creatinine 0.98 mg/dL [0.84 to 1.20 mg/dL], median AST 20 IU/L [17 to 25 IU/L], and median ALT 18 IU/L [15 to 24 IU/L] (Supplementary Table S1 and Supplementary Figures S1 and S2).

### 2.2. Serum Cox-1 and TxB_2_ Values

Median serum Cox-1 value was 11.93 ng/mL [9.65 to 16.08 ng/mL] and median serum TxB_2_ value was 504.72 ng/mL [325.11 to 733.03 ng/mL] (Supplementary Table S1).

**Figure 1:**
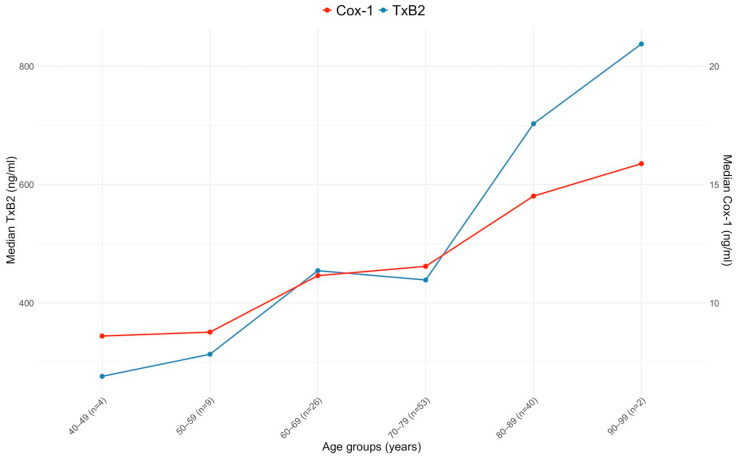
Serum Cox-1 and TxB2 values according to age groups. Cox-1, cyclooxygenase-1; TxB2, thromboxane B2.

Median serum Cox-1 and TxB_2_ values progressively increased within the 10-year age groups (Figure 1 and Supplementary Table S2).

The Kruskal–Wallis test revealed a statistically significant difference among the age groups for both serum Cox-1 and TxB2 values (Supplementary Table S3). The results of Dunn’s test were reported in Supplementary Table S4.

### 2.3. Correlation and Mediation Analysis

**Figure 2:**
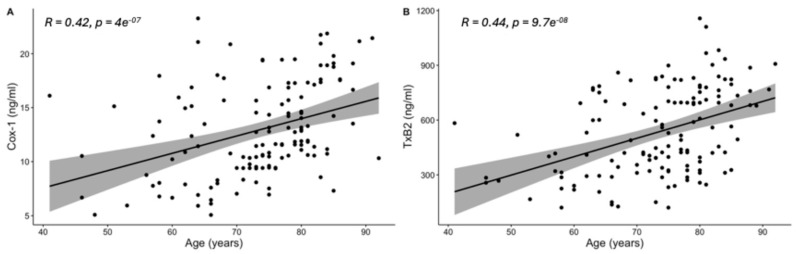
Spearman correlation between (**A**) age and serum Cox-1 and between (**B**) age and serum TxB2. Cox-1, cyclooxygenase-1; TxB2, thromboxane B2. Gray shaded regions represent 95% confidence intervals of the corrrelation lines.

**Figure 3:**
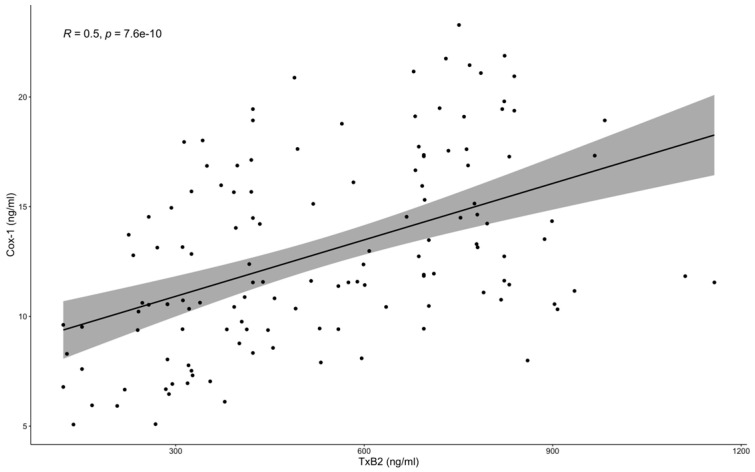
Spearman correlation between serum Cox-1 and serum TxB2. Cox-1, cyclooxygenase-1; TxB2, thromboxane B2. Gray shaded regions represent 95% confidence intervals of the corrrelation line.

A positive and significant correlation has been found between age and Cox-1 (R = 0.42, *p*-values < 0.01) and between age and TxB2 values (R = 0.44, *p*-value < 0.01) (Figure 2A,B). Furthermore, a positive and significant correlation has been found between serum Cox-1 and TxB2 (0.5, *p*-value < 0.01) (Figure 3).

In the first-stage robust regression model, age was positively associated with serum Cox-1 levels (β = 0.22, bootstrap SE = 0.055, z = 3.98, *p* < 0.001), explaining approximately 21% of the variability in Cox-1. In the second-stage model, both Cox-1 (β = 23.52, bootstrap SE = 4.12, z = 5.70, *p* < 0.001) and age (β = 5.67, bootstrap SE = 2.00, z = 2.84, *p* = 0.004) were independently associated with TxB2 levels. The total effect of age on TxB2 was significant (β = 10.87, bootstrap SE = 1.93, z = 5.64, *p* < 0.001). After accounting for Cox-1, the direct effect of age on TxB2 remained significant but was attenuated (β = 5.67, bootstrap SE = 2.00, z = 2.84, *p* = 0.004). The indirect effect of age on TxB2 through Cox-1 was significant based on robust bootstrap estimation (β = 5.20, 95% CI 2.29–8.82), consistent with partial mediation and accounting for approximately half of the total effect. Robust regression diagnostics indicated no influential outliers, and results were stable across 5000 bootstrap replications.

### 2.4. Platelet Cox-1 Expression and TxB2 Production

In vitro experiments were performed in a randomly selected subpopulation (n = 20; 10 patients aged <65 years and 10 patients ≥65 years; 41.3% of female) of patients from our cohort; clinical characteristics of this group were similar to those of the entire population (not shown). In these experiments, we were unable to stratify the population by decade of age due to the limited sample size and we therefore applied an arbitrary age cut-off guided by recent evidence suggesting a diminished efficacy of aspirin therapy in patients over 65 years of age [5,12].

Basal platelet TxB2 production was slightly higher in patients ≥65 years (45 pg/mL) compared to those <65 years (20 pg/mL). Arachidonic acid-induced TxB2 production, measured in the absence of aspirin, was slightly different between the two age groups (182 pg/mL in patients ≥65 years and 178 pg/mL in patients <65 years).

**Figure 4:**
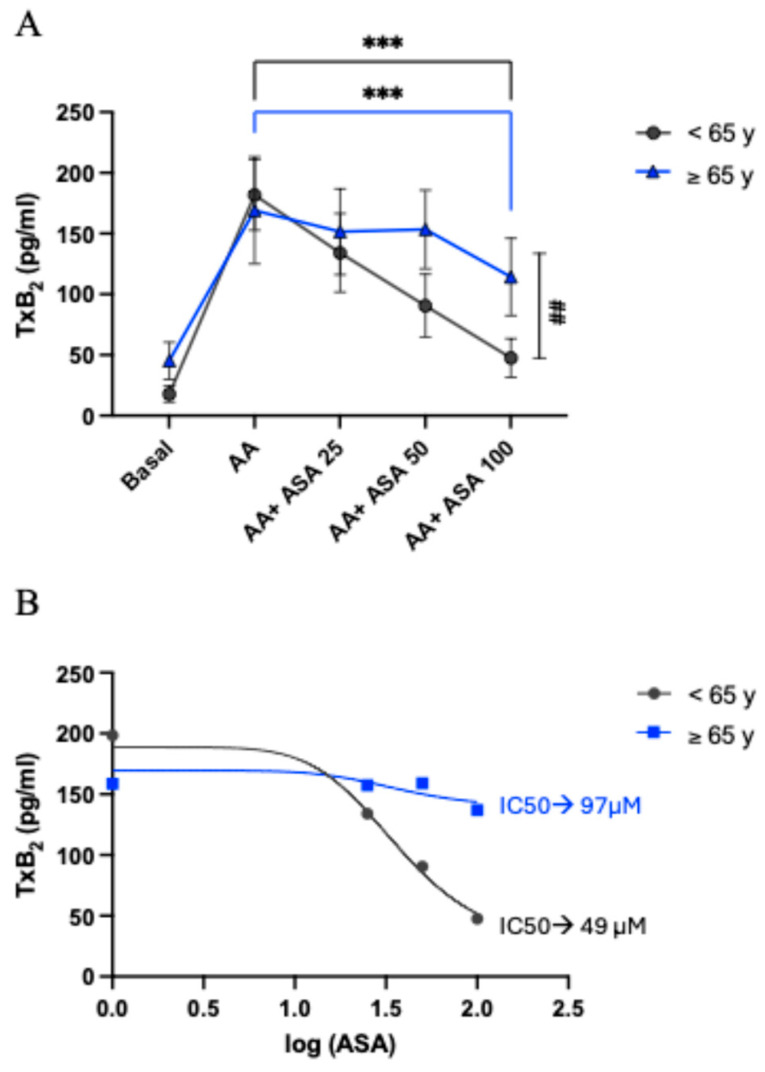
Effect of aspirin on platelets’ thromboxane B2 production. (**A**) Platelets’ TxB2 production was measured after arachidonic acid (AA) stimulation alone or with scalar concentrations of aspirin (25 µM, 50 µM, and 100 µM); *** *p* < 0.001; ## *p* < 0.05. (**B**) Aspirin half-maximal inhibitory concentration for platelets’ TxB2 production. Student’s *t*-test has been used to obtain *p*-values. ASA, aspirin; IC50, half-maximal inhibitory concentration; TxB2, thromboxane B2; y, years.

Arachidonic acid-induced platelet TxB2 production was slightly modified by increasing concentration of aspirin (151, 153, and 135 pg/mL at 25, 50, and 100 µM, respectively; Figure 4A, blue line) in patients ≥65 years, with an IC_50_ of 97 µM (Figure 4B). In contrast, arachidonic acid-induced TxB2 production decreased progressively and significantly with increasing aspirin concentration (134, 91, and 48 pg/mL at 25, 50, and 100 µM, respectively; Figure 4A, black line) in patients <65 years, with an IC_50_ of 49 µM (Figure 4B). Although higher aspirin concentrations were explored in vitro, TxB2 inhibition reached a plateau at the upper range tested, consistent with near-complete Cox-1 acetylation under these conditions, thereby preventing resolution of additional dose-dependent effects.

**Figure 5:**
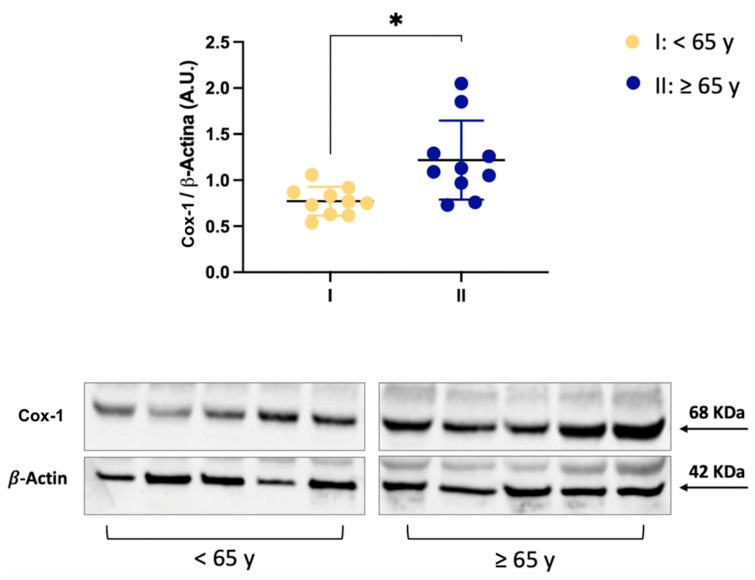
Western blot analysis for platelets’ Cox-1 expression. Patients have been sorted by age groups (i.e., <65 years and ≥65 years). Student’s *t*-test has been used to obtain *p*-values. Cox-1, cyclooxygenase-1. * *p* < 0.05.

Furthermore, we performed platelet Western blot analysis with densitometric quantification, which revealed an increased Cox-1/β-actin ratio in patients ≥65 years compared with those <65 years (Figure 4; *p* < 0.05). β-actin levels were consistent across samples, indicating comparable protein loading (Figure 5).

**Figure 6:**
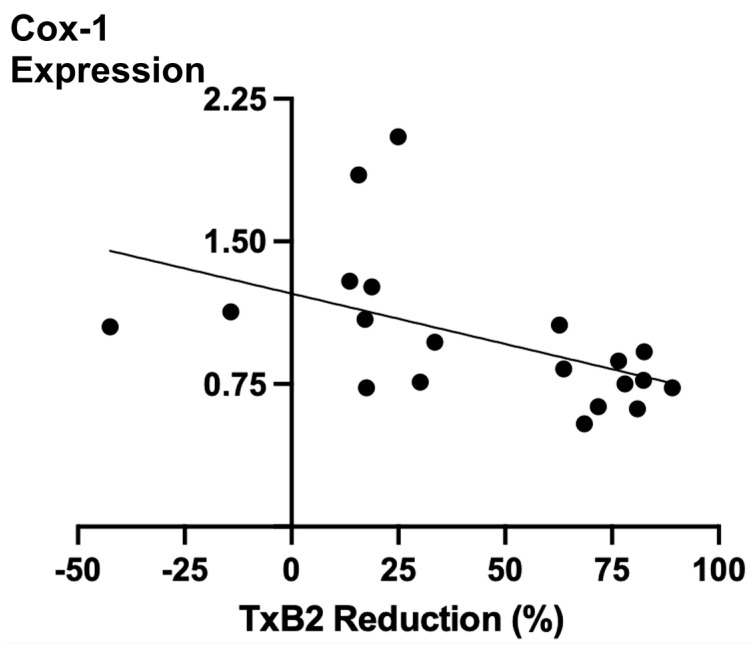
Correlation between platelets’ Cox-1 expression and percentage of reduction in platelets’ thromboxane B2 production by aspirin. Student’s *t*-test has been used to obtain *p*-values. Cox-1, cyclooxygenase-1; TxB2, thromboxane B2.

Finally, we observed a significant negative correlation between Cox-1 protein levels and aspirin-induced platelet TxB2 reduction in patients >65 years (R = −0.640, *p* < 0.01) (Figure 6).

## 3. Discussion

Our findings confirm and extend previous evidence that TxB2 production increases with aging, demonstrating that this age-related overproduction is functionally linked to concomitant platelet Cox-1 upregulation. This mechanistic association is further supported by increased platelet Cox-1 expression, which coincided with a reduced ability of aspirin to inhibit platelet Cox-1 activity in older individuals.

In the present study, we sought to elucidate the mechanisms underlying platelet TxB2 overproduction, previously reported to be closely associated with aging and insufficiently counteracted by concomitant aspirin therapy [9]. The clinical relevance of this phenomenon is underscored by observational data showing that impaired aspirin responsiveness is associated with increased cardiovascular risk [3,10]. To address this hypothesis, we conducted a prospective study in which serum Cox-1 and TxB2 levels were simultaneously measured. Both biomarkers exhibited a parallel age-related increase, suggesting that enhanced TxB2 production in older individuals may be attributable to a concomitant rise in platelet Cox-1 levels; this hypothesis is supported by the fact that at least 95% of serum TxB2 is related to platelet Cox-1 activation [13].

To explore the functional implications of this phenomenon, we assessed aspirin’s inhibitory effect on platelet TxB2 production across age groups and observed a reduced efficacy in patients ≥65 years. Western blot analysis further revealed higher Cox-1 protein expression in these patients, which correlated with the diminished platelet response to aspirin, indirectly indicating enhanced Cox-1 activity in patients over 65 years. Together, these data suggest that age-related increases in serum Cox-1 are functionally relevant, leading to platelet TxB2 overproduction that is not inhibited by aspirin.

The clinical implication of our findings is that aspirin may be less effective in elderly patients compared with younger individuals [6,12,14]. In accordance with our hypothesis, a meta-analysis of randomized trials reported a lack of aspirin efficacy for primary cardiovascular prevention in old patients [12,15] and a recent post hoc analysis of the ASPREE trial reported an absence of aspirin benefit in terms of major adverse cardiovascular events protection, compared to placebo, in elderly patients [5]. These clinical findings, together with the elevated TxB2 levels observed in older patients irrespective of aspirin use, support the hypothesis that additional sources of inter-individual variability—beyond genetic factors or aspirin bioavailability—contribute to the diminished platelet response to aspirin [3,9,10,16,17,18,19].

The aspirin resistance phenomenon, defined as failure of aspirin to completely inhibit platelet Tx production on platelet function tests—laboratory resistance—or to reduce the risk of cardiovascular events—clinical resistance [20]—is of utmost importance in relation to the large numbers of patients who actually receive an indication to aspirin mono or combination therapy, even in the setting of atrial fibrillation [21,22,23]. In this scenario, our study provides new insight into and a better understanding of the mechanism underlying the aspirin resistance phenomenon in older patients; further studies, however, are necessary to validate our data and to identify the mechanism accounting for the Cox-1 upregulation in aging populations.

Our study has some limitations that warrant discussion. First, due to the study design, we were unable to assess the association between the biomarkers examined and future cardiovascular events; nevertheless, prior studies have yet demonstrated a strong relationship between TxB2 levels and cardiovascular outcomes [9,10]. Second, blood samples were not collected longitudinally, and we therefore cannot exclude temporal variability in TxB2 levels. Third, as this was a single-center study conducted in a specific patient subgroup, the generalizability of our findings to the broader population may be limited. Fourth, we did not investigate the molecular mechanisms driving Cox-1 overexpression in older individuals, an important question for future research. Fifth, we did not directly measure the functional activity of Cox-1 that was indirectly assessed by in vitro experiments with aspirin. Finally, certain laboratory and clinical parameters—such as leukocyte counts, body mass index, physical activity, and chronic inflammatory diseases—were not available for analysis. Given that these variables may contribute to systemic serum TxB2 and Cox-1 levels, their potential influence on the observed results should be considered when interpreting these findings.

## 4. Materials and Methods

This study is reported in accordance with the Strengthening the Reporting of Observational Studies in Epidemiology (STROBE) statement for observational studies. The study protocol was approved by the local ethical board of Sapienza University of Rome (Rif. 1306/2007; approved on November 2007), and was conducted according to the principles of the Declaration of Helsinki. All patients provided written informed consent before being included in the study.

### 4.1. Patients and Study Characteristics

Patients with atrial fibrillation followed at the Atherothrombosis Center of the Department of Clinical and Cardiovascular Sciences of Sapienza University of Rome were prospectively included from January 2022 to January 2023 if they had a diagnosis of non-valvular atrial fibrillation and if they consented to participate in the study. Exclusion criteria included age < 18 years, lack of relevant clinical data, lack of blood sample, and refusal to participate in the study.

The following data were collected in an electronic dataset: demographic characteristics (e.g., age, sex category), cardiovascular risk factor (e.g., arterial hypertension, diabetes mellitus), history of cardiovascular disease (e.g., myocardial infarction, ischemic stroke, peripheral arterial disease), thrombotic and bleeding risk scores (i.e., CHA_2_DS_2_-VA and HAS-BLED scores), and type of antithrombotic therapy.

### 4.2. Laboratory Analyses

#### 4.2.1. Serum Preparation

A blood sample was collected from each patient at inclusion in the morning after overnight fasting. Samples were collected in BD Vacutainers without anticoagulants and immediately incubated at 37 °C for 1 h to allow clot formation and platelet activation, leading to TxA2 generation and its subsequent conversion into the stable metabolite TxB2 [13]. Subsequently, samples were centrifuged at 300× *g* for 10 min at room temperature to obtain serum. Serum samples were then aliquoted and stored at −80 °C until analysis.

#### 4.2.2. Platelet Preparation

Citrated blood samples were centrifuged for 15 min at 180× *g* at room temperature to obtain platelet-rich plasma (PRP). To avoid leukocyte contamination, only the top 75% of the PRP was collected. PRP was pre-incubated (20 min at 37 °C) with or without scalar concentrations of aspirin (25, 50, and 100 µM; Sigma-Aldrich, St. Louis, MO, USA) and then stimulated with arachidonic acid (0.5 mM; Mascia Brunelli, Milan, Italy) for 10 min at 37 °C under stirring conditions. Platelet pellets were obtained by centrifuging all samples for 3 min at 3000 rpm. For Western blot analysis of platelet Cox-1, basal platelet pellets (i.e., non-stimulated and non-aspirin-treated) were suspended in Radio-Immunoprecipitation Assay (RIPA) buffer containing protease and phosphatase inhibitor cocktail (10 μg/mL; Thermo Fisher Scientific, Waltham, MA, USA), centrifuged at 10,000× *g* for 20 min at 4 °C to remove insoluble residues, and the supernatants were collected for protein quantification by the Bradford assay. Supernatants and pellets from stimulated samples were stored at −80 °C for analysis of TxB2 and Cox-1 expression.

#### 4.2.3. Serum and Platelet TxB2 Determination

For TxB2 determination in serum (see above) and supernatant of arachidonic acid- stimulated platelets (see above), a commercial ELISA kit (Cusabio, Houston, TX, USA) was used according to the manufacturer’s instructions. Values were expressed as ng/mL. The intra- and inter-assay coefficients of variation for TxB2 were <8% and <10%, respectively.

#### 4.2.4. Serum Cox-1 Determination

Serum Cox-1 (PTGS1) levels were quantified using a human Cox-1 sandwich ELISA kit (LSBio, LS-F22598) according to the manufacturer’s instructions. Serum samples were obtained as previously described for thromboxane determination, allowing whole blood to clot for 1 h at 37 °C before centrifugation. Serum samples were then loaded onto antibody-precoated wells. Following sequential incubation with a biotinylated detection antibody, HRP-conjugated reagent, and TMB substrate, absorbance was measured at 450 nm. Cox-1 concentrations were calculated from a standard curve and expressed in ng/mL. The intra- and inter-assay coefficients of variation were <6.31% and <5.16%, respectively.

The assay employed in this study was designed to quantify serum Cox-1 protein (antigen) rather than enzymatic activity.

To assess if serum Cox-1 was closely related to platelet Cox-1, serum and platelet lysates from healthy samples (n = 15) were normalized to 100 µg total protein (quantified by the Bradford method). Platelet lysates were prepared as described above, thus suspended in RIPA buffer with protease and phosphatase inhibitors, centrifuged at 10,000× *g* for 20 min at 4 °C, and supernatants were collected. Samples containing 100 µL of normalized serum or lysate were loaded onto the ELISA wells and processed as above described. A significant correlation was observed between serum and platelet lysate Cox-1 levels (Pearson’s r = 0.607, *p* < 0.01).

#### 4.2.5. Platelet Cox-1 Expression

For Western blotting analysis, platelet lysates (prepared as detailed above) with equal protein amounts (30 µg/lane) were solubilized in 4X Laemmli buffer containing 2-mercaptoethanol and loaded onto denaturing SDS 10–12% polyacrylamide gels. Membranes were incubated overnight at 4 °C with rabbit polyclonal anti-Cox-1 (Abcam, Cambridge, UK) or mouse monoclonal anti-β-actin (Santa Cruz Biotechnologies, Dallas, TX, USA) primary antibodies, followed by an HRP-conjugated secondary antibody (1:3000; Bio-Rad, CA, USA) for 1 h. Immune complexes were detected using enhanced chemiluminescence substrate (ECL; Bio-Rad, Hercules, CA, USA). Densitometric analysis of bands was performed using ImageJ software (version 1.54), and values were expressed in arbitrary units (A.U.).

### 4.3. Statistical Analysis

Baseline characteristics of the included patients are reported as descriptive statistics. Categorical variables were reported as counts and percentages. Continuous variables were reported as mean and standard deviation (SD) or median and interquartile range (IQR) values, according to data distribution after applying the Shapiro–Wilk test. Categorical variables were compared using the chi-squared or Fisher’s exact tests and continuous variables using Student’s *t* test or the Mann–Whitney U test, as appropriate. Serum Cox-1 and TxB2 levels were stratified into 10-year age groups and compared using either ANOVA or the Kruskal–Wallis test, as appropriate. When the overall test was significant, post hoc pairwise comparisons were conducted using Dunn’s test with Bonferroni correction. Correlation test was used to test the association between age and TxB2 values, between age and Cox-1, and between Cox-1 and TxB2. Furthermore, a robust mediation analysis was conducted to examine whether Cox-1 mediates the relation between age and TxB2. The in vitro study was performed in a randomly selected subpopulation of patients from our cohort that have been sorted by age groups (i.e., <65 years and ≥65 years). In these experiments, we applied an arbitrary age cut-off guided by recent evidence suggesting a diminished efficacy of aspirin therapy in patients over 65 years of age [12,24,25]. Statistical significance was set at 0.05. RStudio (version 2025.09.2+418, R Core Development Team, Vienna, Austria) was used for the analysis.

## 5. Conclusions

Aging is associated with upregulation of platelet Cox-1 coincidentally with overproduction of platelet TxB2, suggesting that in older populations, platelet Cox-1 functionality is enhanced. Aspirin’s inability to inhibit Cox-1 in older populations provides a mechanistic insight into recent study showing that aspirin is clinically ineffective in the older population. Further studies are warranted to elucidate the mechanisms underlying the increased platelet Cox-1 upregulation by aging.

## Figures and Tables

**Table 1:** Baseline characteristics of patients included in primary analysis.

*Variables*	n = 134
*Median age, years (IQR)*	75 [67–80]
*Female sex, n (%)*	58 (43.3)
***Medical history, n (%)***	
* Arterial hypertension*	95 (89.6)
* Diabetes mellitus*	41 (38.7)
* Dyslipidemia*	68 (51.5)
* Former smokers*	13 (12.3)
* Established coronary disease*	16 (15.1)
* Cerebrovascular disease*	20 (18.9)
* Carotid atherosclerotic disease*	34 (32.1)
***Risk scores and lab values***	
* CHA_2_DS_2_-VA, [IQR]*	3 [2–4]
* HAS-BLED, [IQR]*	1 [1–1]
* Cox-1, ng/mL [IQR]*	12 [10–16]
* TxB2, ng/mL [IQR]*	505 [325–733]
***Medical therapies, n (%)***	
* Apixaban*	49 (36.6)
* Dabigatran*	21 (15.7)
* Edoxaban*	18 (13.4)
* Rivaroxaban*	15 (11.2)
* Warfarin*	31 (23.1)

IQR, interquartile range; TxB2, thromboxane B2.

## Data Availability

The data underlying this article are available in the article and in its online Supplementary Materials.

## Supplementary Materials

The following supporting information can be downloaded at: https://www.mdpi.com/article/10.3390/ijms27114972/s1.
