# Supplementary material for: Age-Related Platelet Cox-1 Upregulation in Atrial Fibrillation
Source: Int J Mol Sci. 2026 May 30;27(11):4972. doi: 10.3390/ijms27114972 (PMC13256525; doi:10.3390/ijms27114972)

Supplementary Table S1. Results of Shapiro-Wilk normality test

Variable	p-values
Age	9.288e-05
Cox1-1	0.006675
TxB_2	0.0007792
Hemoglobin	0.1496
Platelets	0.002739
Creatinine	4.724e-13
AST	5.392e-10
ALT	1.7e-10

Supplementary Table S2. Median serum Cox-1 and TxB_2 values according to age groups

Age Group (years)	Median Serum Cox-1 (ng/ml) [IQR]*	Median Serum TxB_2 (ng/ml) [IQR]*
40–49	8.61 [6.28 – 11.92]	276.11 [265.22 – 358.95]
50–59	8.77 [7.77 – 13.72]	313.40 [225.24 – 401.32]
60–69	11.16 [7.68 – 15.88]	454.67 [290.25 – 700.25]
70–79	11.55 [9.52 – 14.34]	438.90 [349.72 – 694.90]
80–89	14.52 [11.89 – 17.92]	702.90 [446.90 – 802.50]
90–99	15.89 [13.10 – 18.69]	837.73 [802.75 – 872.71]

Supplementary Table S3. Results of Kruskal–Wallis test

Variables	Chi-squared	df	p-values
COX-1	18.964	5	0.001952
TxB_2	26.975	5	5.768e-05

Supplementary Table S4. Results of Dunn's test

Variables	Comparison	Z	P unadjusted	P adjusted (Bonferroni)
COX-1	40–49 (n=4) - 50–59 (n=9)	-0.40717441	0.6838798950	1.00000000
	40–49 (n=4) - 60–69 (n=26)	-1.01812023	0.3086208208	1.00000000
	50–59 (n=9) - 60–69 (n=26)	-0.78122781	0.4346685205	1.00000000
	40–49 (n=4) - 70-79 (n=53)	-1.09605452	0.2730549180	1.00000000
	50–59 (n=9) - 70-79 (n=53)	-0.89771607	0.3693369385	1.00000000
	60–69 (n=26) - 70-79 (n=53)	-0.08984925	0.9284070091	1.00000000
	40–49 (n=4) - 80-89 (n=40)	-2.44099752	0.0146467553	0.21970133
	50–59 (n=9) - 80-89 (n=40)	-2.80644516	0.0050091438	0.07513716
	60–69 (n=26) - 80-89 (n=40)	-2.91070191	0.0036061788	0.05409268
	70-79 (n=53) - 80-89 (n=40)	-3.39818417	0.0006783474	0.01017521
	40–49 (n=4) - 90-99 (n=2)	-1.35318776	0.1759956540	1.00000000
	50–59 (n=9) - 90-99 (n=2)	-1.18609563	0.2355845347	1.00000000
	60–69 (n=26) - 90-99 (n=2)	-0.85183595	0.3943051510	1.00000000
	70-79 (n=53) - 90-99 (n=2)	-0.83790467	0.4020842462	1.00000000
	80-89 (n=40) - 90-99 (n=2)	0.14929557	0.8813204070	1.00000000
TxB_2	40–49 (n=4) - 50–59 (n=9)	0.08751235	9,30E+05	1.00000000
	40–49 (n=4) - 60–69 (n=26)	-130.086.044	1,93E+05	1.00000000

	50–59 (n=9) - 60–69 (n=26)	-194.252.055	5,21E+04	0.7811117242
	40–49 (n=4) - 70-79 (n=53)	-160.189.253	1,09E+05	1.00000000
	50–59 (n=9) - 70-79 (n=53)	-244.977.943	1,43E+04	0.2144156219
	60–69 (n=26) - 70-79 (n=53)	-0.55107403	5,82E+05	1.00000000
	40–49 (n=4) - 80-89 (n=40)	-270.823.060	6,76E+03	0.1014645072
	50–59 (n=9) - 80-89 (n=40)	-399.204.978	6,55E+01	0.0009825692
	60–69 (n=26) - 80-89 (n=40)	-286.418.778	4,18E+03	0.0627120122
	70-79 (n=53) - 80-89 (n=40)	-281.497.460	4,88E+03	0.0731716339
	40–49 (n=4) - 90-99 (n=2)	-256.155.402	1,04E+04	0.1563075498
	50–59 (n=9) - 90-99 (n=2)	-290.502.081	3,67E+03	0.0550843021
	60–69 (n=26) - 90-99 (n=2)	-207.099.755	3,84E+04	0.5753853808
	70-79 (n=53) - 90-99 (n=2)	-192.656.115	5,40E+04	0.8105151549
	80-89 (n=40) - 90-99 (n=2)	-110.156.925	2,71E+05	1.00000000

Supplementary Figure S1. Median platelets values according to age groups

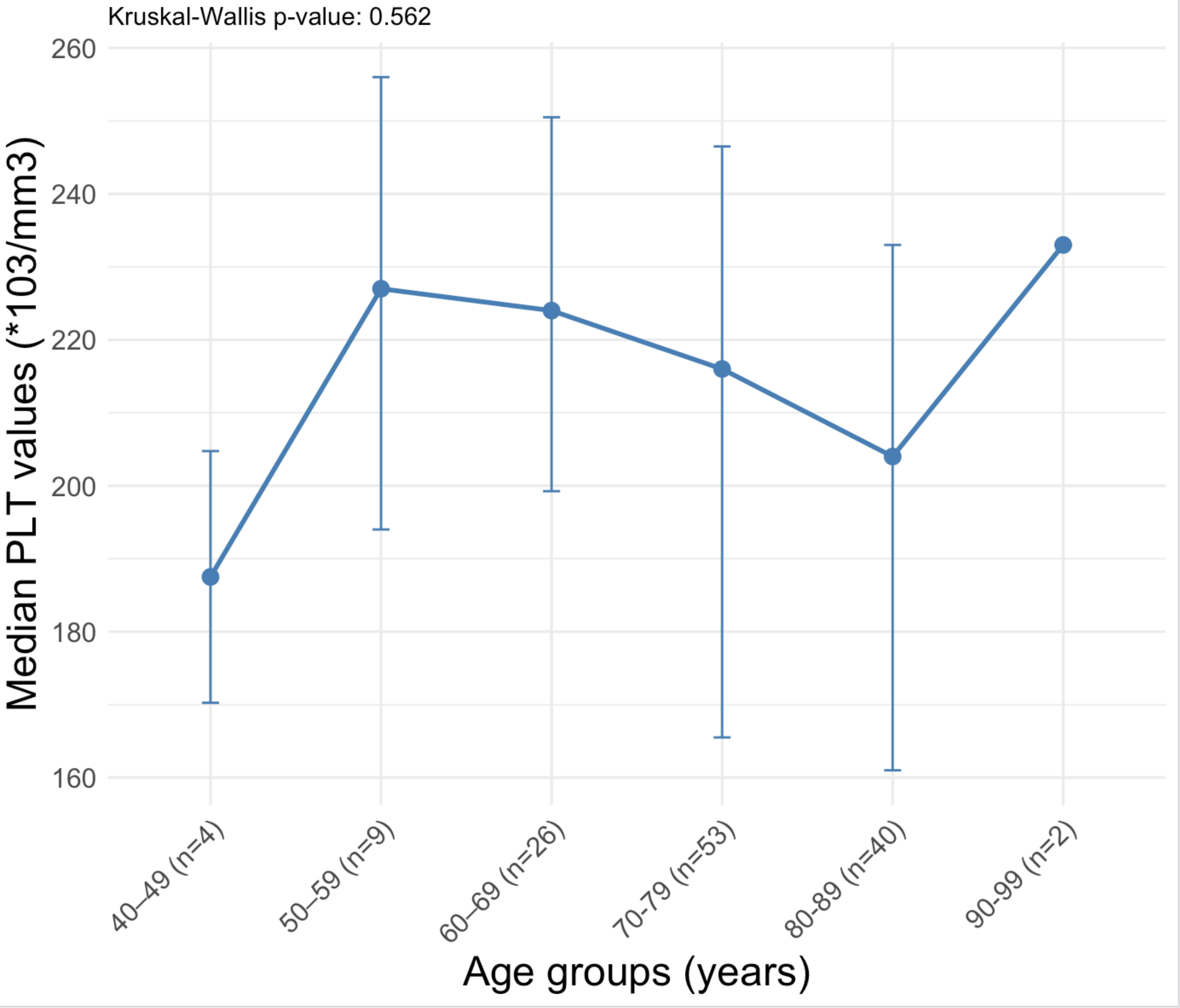

Supplementary Figure S2. Prevalence of diabetes mellitus according to age groups

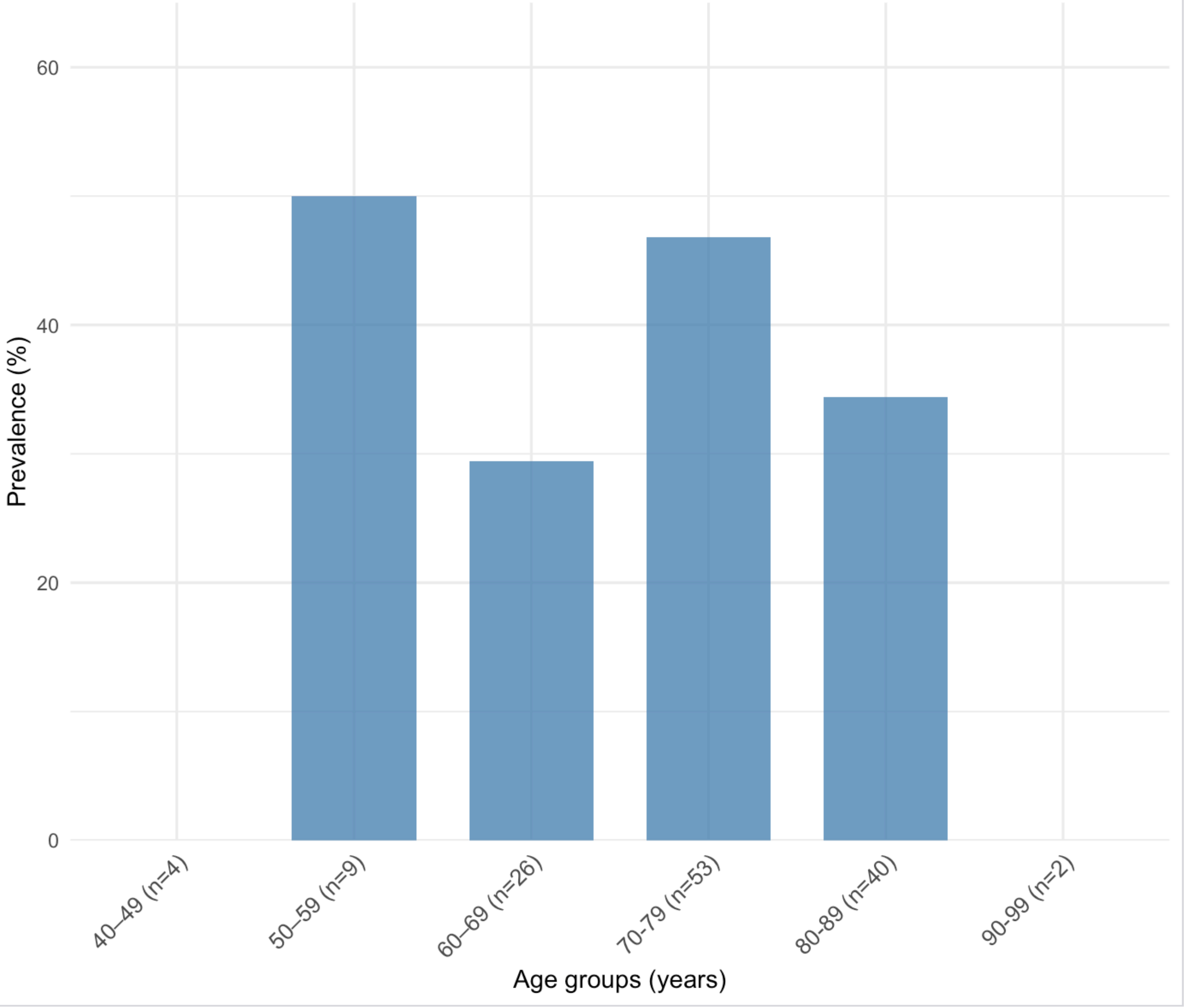

Supplement: Supplementary file 1 [file ijms-27-04972-s001.zip › ijms-4261961-supplementary.pdf]
